# Differential Expression of Serotonin, Tryptophan Hydroxylase and Monoamine Oxidase A in the Mammary Gland of the *Myotis velifer* Bat

**DOI:** 10.1371/journal.pone.0075062

**Published:** 2013-09-24

**Authors:** Cristián Vela Hinojosa, Miguel Angel León Galván, Miguel Tapia Rodríguez, Gerardo López Ortega, Marco Antonio Cerbón Cervantes, Carmen Adriana Mendoza Rodríguez, Patricia Padilla Cortés, Luis Antonio Martínez Méndez, Francisco Javier Jiménez Trejo

**Affiliations:** 1 Faculty of Chemistry, Universidad Nacional Autónoma de México, México D.F., Mexico; 2 Department of Biology, Universidad Autónoma Metropolitana-Iztapalapa, México D.F., Mexico; 3 Microscopy and HPLC Units, Instituto de Investigaciones Biomédicas, Universidad Nacional Autónoma de México, México D.F., Mexico; Clermont-Ferrand Univ., France

## Abstract

The mammary gland has long drawn the attention of the scientific community due to the limited knowledge of some fundamental aspects involved in the control of its function. *Myotis velifer*, a microchiropteran species, provides an interesting model to study some of the regulatory factors involved in the control of the mammary gland cycle. Having an asynchronous, monoestrous reproductive pattern, female *M. velifer* bats undergo drastic morphological changes of the breast during the reproductive cycle. Current research on non-chiropteran mammals indicates that serotonin (5-HT) plays a major role in the intraluminal volume homeostasis of the mammary gland during lactation; however, an analysis of both the expression and localization of the main components of the serotonergic system in the bat mammary gland is lacking. Thus, the objectives of the present study were: to describe the gross and histological anatomy of the mammary gland of *M. velifer* to establish the lactation period for this species; to analyze the distribution and expression of the main serotonergic components in the mammary tissues of these bats under the physiological conditions of lactation, involution and the resting phase; and to provide information on the involvement of 5-HT in the regulation of the physiological function of this organ. To assess the expression and localization of serotonergic components, multiple immunofluorescence, Western blot and HPLC methods were used. 5-HT and the enzyme that catalyzes its synthesis (TPH) were located in both myoepithelial and luminal epithelial cells, while the enzyme responsible for the catabolism of this neurohormone (MAO A) was found in luminal epithelial cells as well as in secreted products. We also found an increased expression of serotonergic components during lactation, indicating that elements of the serotonergic system may play an important role in lactation in this species of bat in a way similar to that of other mammal species.

## Introduction

The mammary gland is regarded as the main distinguishing feature that gives mammals their name. It is responsible for producing milk as the only food source that provides nutrition to offspring in the early stage of life. It is an exocrine gland and a modified sweat gland [1]. Complex in its development and regulation, the mammary gland is one of the few tissues in mammals that can undergo repeated growth, functional differentiation, and regression [2].

The mammary gland is comprised of parenchymal structures called alveoli that invade the mammary fat pad, from which milk is expelled through the lactiferous duct to the nipple. These structures are composed of basal myoepithelial cells and the secretory luminal epithelium. The alveoli are considered the primary morphological structure of the mammary gland, and undergo differentiation that results in the terminal differentiation of the gland [3].

Lactation is a complex physiological condition that often requires a larger energy supply than pregnancy and induces a series of adaptations in the physiology of the female, including an increase of maternal brain plasticity [4]. After lactation, the gland goes through a process of apoptotic cell death and remodeling known as involution and often described as a two-step process because of its reversibility [4], [5]. Mammary gland development is controlled by the dynamic interplay of endocrine hormones and locally-produced factors [6]. Prolactin is essential for initiating and maintaining lactation, while oxytocin is important for milk ejection [7].

In recent years, studies have shown that serotonin (5-hydroxytriptamine; 5-HT) regulates several biological processes outside the central nervous system (CNS), including reproductive and endocrine functions [8]–[11]. With respect to the mammary gland, a major role has been proposed in regulating intraluminal volume homeostasis, a common physiological phenomenon for fluid-secreting organs that consists in compartmentalization and volume regulation of specialized fluids in relation to demand and the physical space available in the organ [6], [12]. 5-HT regulates volume homeostasis in an autocrine-paracrine manner in the mouse mammary gland_,_ and its biosynthesis is induced by milk stasis (accumulation) during lactation. Similar observations have been made in bovines and humans [6], [12]. 5-HT was found to have a disruptive action on the tight junctions that are responsible for the compartmentalization of milk secretion. It also activates 5-HT_7_ receptors which create a biphasic response that, accompanied by increased intraluminal pressure, initially strengthens tight junctions but upon sustained exposure causes their disruption and induces mammary gland involution [12], [13].

Tryptophan hydroxylase (TPH) and monoamine oxidase (MAO A) are two well-known enzymes involved in the metabolism of 5-HT by mediating its biosynthesis and catabolism, respectively. It has been demonstrated that once the mammary gland is stimulated by prolactin, it expresses genes essential for serotonin biosynthesis [6]. Interestingly, TPH mRNA is elevated during pregnancy and lactation [6].

*Myotis velifer* (J. A. Allen, 1890) is a microchiropteran, vespertilionid species that lives at lower elevations in the southwestern United States from Kansas to southern Nevada and southeastern California, and southward through Mexico to Honduras, encompassing temperate environments and arid and semi-arid zones [14]–[17]. It is a small, insectivorous species with a body mass between 7.5 and 12 g. Its gestation lasts 50 to 60 days and lactation can continue for up to 60 days [15], [18]. Interestingly, this species has a seasonal, asynchronous monoestrous reproductive cycle [19]. Mating occurs mainly during late autumn but also in winter, when males occasionally wake up from torpor [15], [20], [21]. The sperm are stored in the genital tract (uterus and utero-tubal junction) of the female after insemination; thus, ovulation is delayed for several weeks and fertilization and gestation occur only after the hibernation season. This mechanism ensures that pups are born in the most favorable season (spring), when both good food availability and weather are present [19]. Although it has not been demonstrated, it is suggested that bat populations of *M. velifer* in central Mexico move to higher altitudes in the autumn to perform mating and hibernation, and then return to the shelters of lower altitude in late winter for gestation, births and lactation [22].

Interpretations of the reproductive status of bats based on nipple size alone can be ambiguous; however, a combination of criteria, including relative nipple size, the presence or absence of hairs on the nipple, and the degree of nipple cornification, makes it possible to determine the relative stages of lactation and involution [23], [24].

Like most bats, *M. velifer* possesses a pair of mammary glands in the axillary position that experience a marked development which establishes the lactation season, and then an abrupt involution that leads to a resting phase when the gland remains inconspicuous and resembles the prepubertal condition [25]. Externally, the enlarged mammary glands of the lactating phase show prominent nipples and all hair disappears from this area [26]. The mass of the mammary gland is reported to be substantial during lactation: in species like *Tadarida brasiliensis* it represents 8% of female post-absorptive body mass [27].

The present study describes, for the first time, the anatomical changes of the mammary gland of *M. velifer* during the reproductive cycle, in order to determine the lactation period for this microchiropteran species from central Mexico. It also assesses the presence, distribution and expression of serotonergic components (TPH, MAO A and 5-HT) in the mammary gland under the conditions of lactation, involution and the resting phase, to provide information related to the possible involvement of 5-HT in the physiological regulation of this gland.

## Materials and Methods

### Ethics Statement

*Myotis velifer* is not listed as an endangered species in the *Norma Oficial Mexicana* NOM-059-ECOL-2010 for the protection of native wild species of Mexico [28]. Indeed, this species is listed as ‘of least concern’ by the IUCN because of its wide distribution and presumed large population [29]. All protocols for the capture and handling of the live animals, as well as the euthanasia procedure used in this study, were conducted in strict accordance with the guidelines established by the American Society of Mammalogists for the use of wild mammals in research [30], and were approved by the Ethics Committee of our institution. The study area is located on federal land so permission FAUT-024 for collecting wild fauna was issued to Dr. Ricardo López-Wilchis, Director of the Laboratory of Mammal Biology and Ecology at the UAM-I campus, by the *Dirección General de Vida Silvestre de México* (an agency of SEMARNAT).

### Animal Capture and Processing

Adult females of *M. velifer* were captured during May, July and August 2012 in a shelter in central Mexico where bats were observed to perform lactation (Chicomostoc cave, 19°57′54″N, 97°36′09″W, 1,420 m elevation). The bats were captured during the evenings using harp traps placed at the entrance of the cave. Traps were checked every 15 minutes for 2 hours using red light [31]. The animals were sexed and only adult females were selected and then placed individually in cotton bags. The criteria for adulthood were evaluated in accordance with [32]–[34]. The remaining individuals were released immediately. The selected animals were taken to a laboratory set up in a village near the cave and processed five hours after their capture. The condition of the mammary gland and nipple were determined according to the parameters suggested by [23], [24]: *i.e.*, relative nipple size, the presence or absence of hairs on the skin around the nipple, and the degree of nipple cornification.

For this research, 15 females of *M. velifer* were used. Three groups of 5 bats each were selected as representative of the three key stages of mammary gland development: lactation (May), involution (July) and the resting phase (August).

The animals were euthanized by decapitation, and immediately both mammary glands were dissected, weighed in a Mettler model AB204 balance (±0.01 mg), frozen in 2-methylbutane pre-chilled with dry ice, and stored in cryovials immersed in liquid nitrogen (−170°C) for transport to the laboratory where they were stored at −75°C until further use.

### Histology of the Mammary Gland

In order to perform basic histological description, tissues from the three different stages of the mammary gland cycle: lactation, involution and resting phase were selected and fixed in formalin (10%). Macroscopic characteristics were recorded and the overall diameter of each nipple and mammary gland was measured [23], [24]. Cross-sections (8 µm thick) were cut on a cryostat at −20°C and mounted on gelatin-coated slides. Then sections were stained with hematoxylin-eosin and observed using a light microscope (Leica, model DM2500 P, Leica Microsystems, Nussloch, Germany). Images were obtained with a camera (Q-Color5, Olympus, Tokyo, Japan). The histological features analyzed included the size of the ducts and alveoli, the degree of vascularization and the presence of secretion products.

### Indirect Multiple Immunofluorescence to Localize Serotonin, TPH and MAO A Enzymes in the Mammary Glands

Three mammary glands representative of the three stages of the cycle (lactation, involution, resting phase) were selected and processed by immunofluorescence.

Sections of mammary gland tissue (12 µm thick) were cut on a cryostat, mounted onto gelatin-coated slides, and fixed in 4% paraformaldehyde dissolved in phosphate buffer (PBS 0.1 M, pH 7.4). Tissue preparations were used to detect 5-HT, TPH and MAO A, as described previously [10], [11]. Sections were incubated with the following antibodies: mouse monoclonal anti-5-HT (Genetex Inc. Irvine, California, USA), rabbit anti-TPH and rabbit anti-MAO A (Santa Cruz Biotechnology, Santa Cruz CA, USA) in 1∶100 dilutions overnight at 4°C, followed by the secondary antibodies in 1∶100 dilutions: goat anti-rabbit rhodamine (Temecula, CA, USA) and goat anti-rabbit IgG (HL) (Invitrogen Corporation, CA, USA) The slides were cover-slipped with Dako fluorescence mounting medium (DAKO, Inc., Denmark). The brain of the same *M. velifer* bat was used as control. In control experiments, slides were incubated with pre-immune serum. All experiments were performed in triplicate.

In order to localize TPH and MAO A enzymes in luminal epithelial and myoepithelial cells, the mammary glands from lactating females were dissected, stored in liquid nitrogen, and then processed as described above. Tissues were sectioned at 12 µm, fixed in 4% neutral buffered formalin for 30 minutes, incubated with 1% Triton-X 100 in PBS for 1 h at room temperature, rinsed three times with PBS, blocked for 1 h with 10% goat serum in PBS and then incubated with Alexa fluor 488 conjugated anti-actin C4 (1∶100) and mouse anti-cytokeratin 8 MAB3414 (1∶100) (both from Millipore Corporation, Billerica, MA, USA) during four hours. Sections were rinsed three times with PBS and conjugated with secondary Alexa Fluor 350 goat-anti-mouse antibody (Invitrogen Corporation, CA, USA) for cytokeratin-8 staining, followed by incubation with a second primary antibody, rabbit anti-TPH and rabbit anti-MAO A (1∶100 Santa Cruz Biotechnology, USA). Samples were then incubated with DAPI (2 mg/ml) (Millipore) for 10 min at room temperature. Rat uterus tissues at estrus were included as a positive control.

All tissue sections from the mammary glands processed by immunofluorescence were visualized using an epifluorescence microscope (Nikon E600, Nikon, Melville, NY) and images were acquired with a digital camera (Nikon) adapted to the observation tube of the microscope. Images were digitized and figures processed using Adobe Photoshop software 10.0.1 (Adobe Systems Incorporated, San Jose, CA, USA).

### Immunodetection with Western Blot Against TPH and MAO A Enzymes at Different Lactation Stages

Tissue samples of the mammary glands stored at −75°C were thawed and homogenized in a buffer containing Trizma hydrochloride (Tris-HCl; 0.05 M, pH 7.4), dithiothreitol (1 mmol), and tetraacetic acid (ethylenebis (oxyethylenenitrilo)), and ethylene glycol bis (2-aminoethyl ether) N,N,N′,N′-tetraacetic acid (EGTA) (1 mM), supplemented with a mixture of protease inhibitors (Complete, EDTA-free, Roche-Mannheim, Germany). The amount of aggregated protein was quantified by Bradford assay. Samples (20 µg of protein/well) diluted in Laemmli solution were electrophored under reducing conditions (5% b-mercaptoethanol) through sodium dodecyl sulphate-polyacrylamide gels (12%) at 140–150 V for 2 hours. Pre-stained molecular weight markers (Amersham-Pharmacia-Biotech, Piscataway, NJ, USA) were used to determine the relative mobility of proteins. Following electrophoresis, gels were equilibrated in a buffer containing Tris 25 mmol, glycine 192 mmol and 20% methanol. The proteins were then transferred to nitrocellulose sheets (BIO-RAD) at 276 mA for 2 hours at 4°C.

Membranes were blocked with non-fat milk (5%) dissolved in Tris (20 mmol)-sodium chloride (500 mmol) buffer (TBS) for 2 hours at room temperature. They were then washed with TBS containing Tween-20 (0.05%; Tween-Tris sodium chloride (TTBS), and incubated with rabbit anti-TPH (1∶1500) and goat anti-MAO A (1∶2500; both from Santa Cruz Biotechnology) at room temperature overnight. Membranes were incubated with goat anti-rabbit and donkey anti-goat secondary antibodies conjugated with horseradish peroxidase (1∶5000, Vector) for 2 hours at room temperature. Finally, after washing the membranes with TTBS, peroxidase activity was revealed using a chemiluminescence-based detection kit according to the manufacturer’s protocol (ECL, Amersham-Pharmacia-Biotech, Buckinghamshire, UK). Membranes were exposed to film sheets for 2 minutes at room temperature and the films were then developed (Dektol-19, Kodak, Rochester, NY) and fixed (Rapid Fixer, Kodak). Images of these films were captured, digitized and analyzed by densitometry (with ImageJ v1.46 software). All measurements were corrected based upon the average value of the background of each film.

### Concentration of Serotonin in Mammary Glands Using HPLC

HPLC was performed to detect serotonin and L-tryptophan (L-Trp) concentrations in tissues from the three representative stages of mammary gland development. In order to perform more precise measurements of 5-HT concentrations, we used the protocols from [10], [35]. Tissue samples were homogenized in sodium metabisulfite (4 mmol) in HClO4 (0.85%) solution and centrifuged at 18,000 g for 16 minutes at 4°C. The supernatants were collected and protein concentrations determined. Filtered (0.45-mm acrodiscs) 20-mL supernatant samples were then injected into the HPLC (Waters Corporation and Waters Model 600, 270 nm UV detection, Phenomenex, with a C18 symmetry column, 150×0.45 mm in a reverse phase mode (2000 psi, 1 mL/minute)). The mobile phase was prepared with ammonium acetate (50 mM, pH 3.50: acetonitrile). Chromatograms were recorded online and the peak heights measured using Millennium 32 Software (Waters Co., Milford, Mass). Results are expressed in micrograms of 5-HT/L-Trp per milliliter of sample (µg/mL tissue).

### Statistical Analyses

Statistical comparisons among different groups for Western blot and HPLC data were carried out with a one-way ANOVA (significance level set at p<0.05) followed by a Tukey test.

## Results

### Morphological Description of the Mammary Gland during the Different Lactation Stages

**Figure 1:**
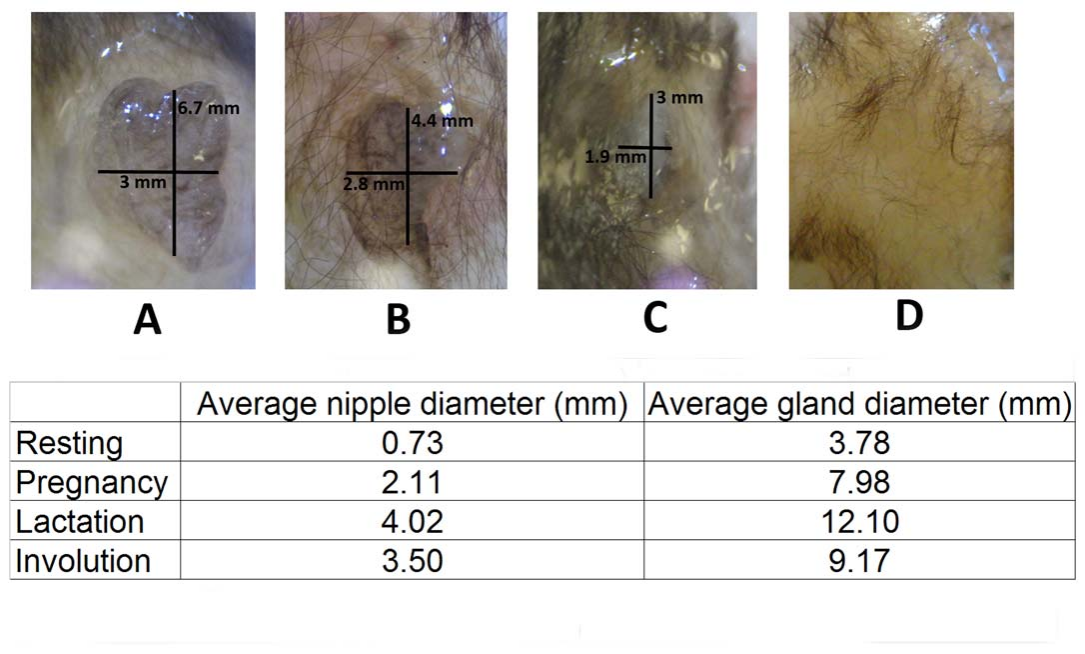
Morphometrical changes in the mammary gland cycle. Images of mammary glands from adult females of *M. velifer* acquired during different stages of development (A, lactation; B, early involution; C, late involution; and D, resting phase). Average measures of the diameter of the mammary gland and nipple during pregnancy, lactation, involution, and the resting phase are summarized in the chart.

We compared the average diameter of the nipple and mammary gland in the three different physiological conditions (Figure 1: A, lactation; B, early involution; C, late involution; and D, resting phase). In the late stages of mammogenesis (prior to parturition) and during early lactation, the nipple occupies most of the gland and looks expanded and hydrated. During late lactation, the nipple is darker and shows evidence of necrosis. In early involution, it shows slight cornification and begins to shrink. In the late involution stage the nipple usually looks whitish and is more difficult to distinguish. Finally, during the resting phase it is inconspicuous and, in most cases, seems to disappear completely.

**Figure 2:**
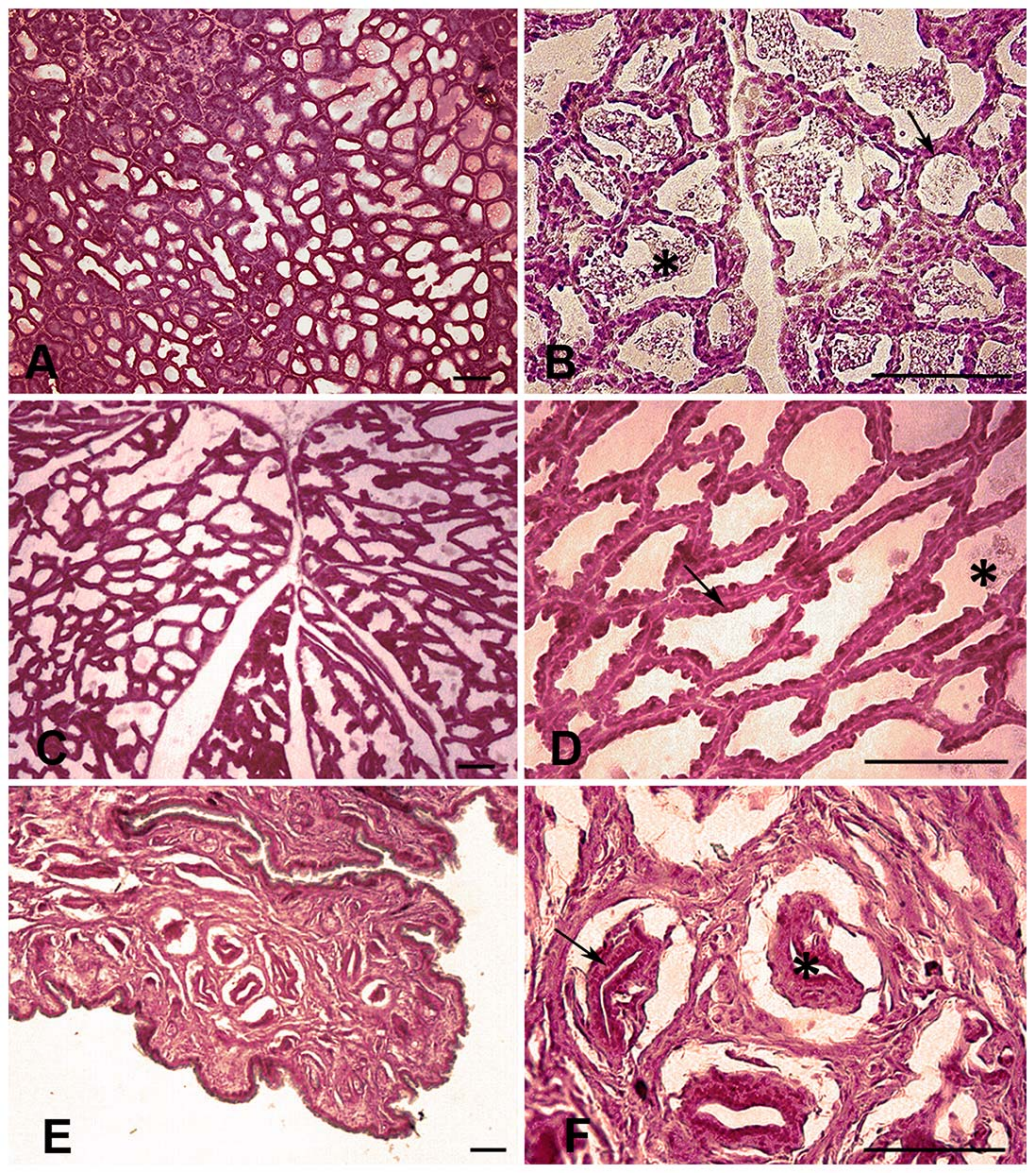
Histology of the mammary gland. Photomicrographs showing the basic histology of the mammary gland of *M. velifer* in three stages: lactation (A and B) featuring secretion products inside the alveoli (asterisk) and epithelial alveolar cells (arrow); involution (C and D), with scarce secretion products inside the alveoli (asterisk) and epithelial alveolar cells (arrow); and the resting phase (E and F), where stroma occupies most of the gland (asterisk) and epithelial alveolar cells are found in a restricted area (arrow). Transverse sections. Scale: 100 µm.

The morphology of the mammary gland was studied by conventional histology. Figure 2 shows the basic histology of the bat mammary gland in three different stages: lactation (2A and 2B) with abundant secretion products inside the alveoli (asterisk) and epithelial alveolar cells (arrow); involution (2C and 2D), with scarce secretion products in the alveoli (asterisk); and the resting phase (2E and 2F), when prominent, dense stromal tissue invades most of the gland and surrounds the alveoli (asterisk), while the epithelial alveolar cells are found only in a restricted area (arrow).

### Variations in the Expression of 5-HT, and TPH and MAO A Enzymes Using Immunofluorescence

**Figure 3:**
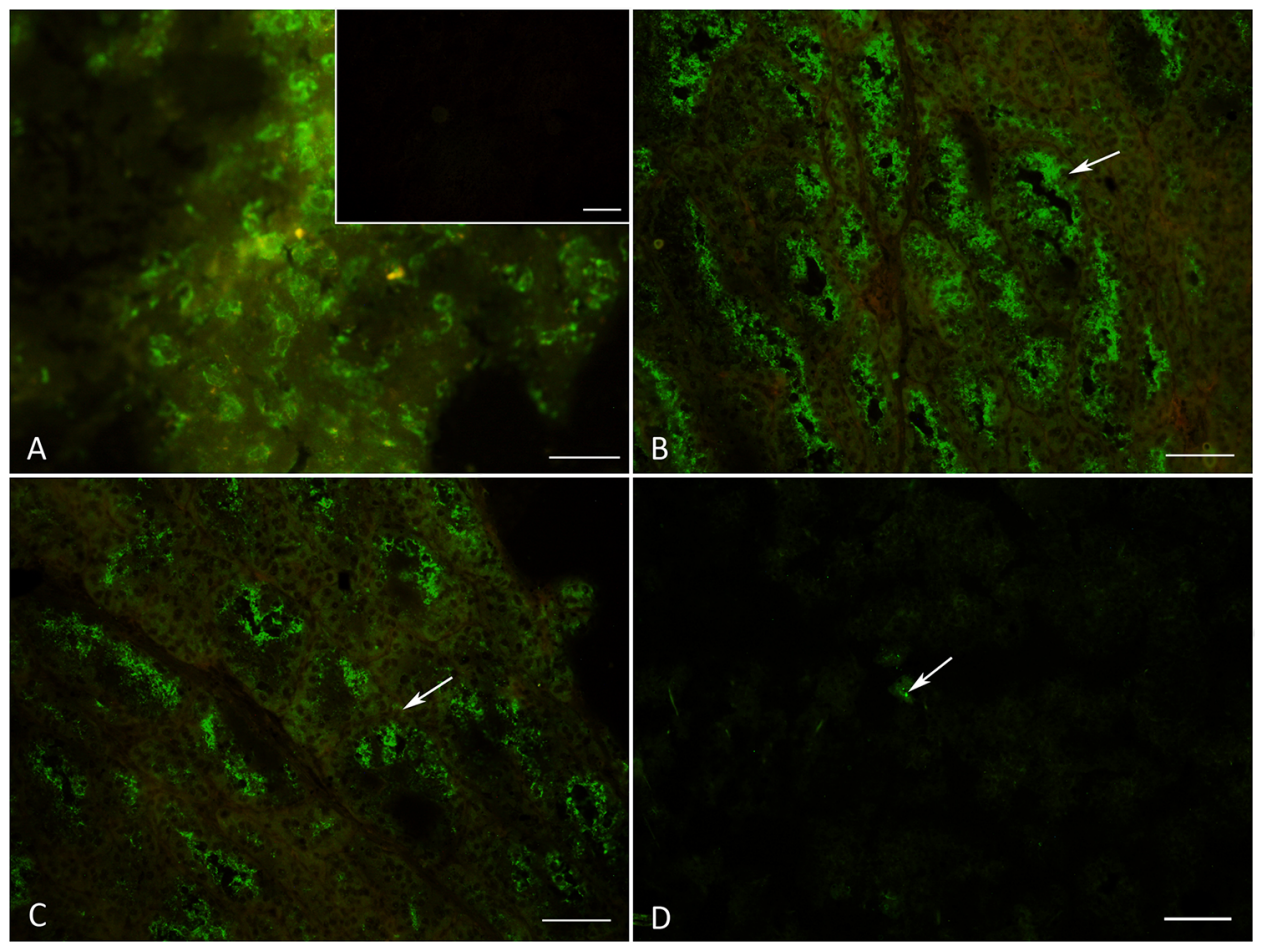
Differential expression of MAO A. Photomicrographs showing MAO A immunostaining. Figure 3A shows brain tissue from *M. velifer* used as a positive control for this technique. The inset shows the mammary gland of *M. velifer* as a negative control when slides were incubated with pre-immune serum. Figures 3B, 3C and 3D show MAO A differential expression during lactation, involution and the resting phase, respectively. Longitudinal sections. Scale: A: 20 µm; B, C, D: 100 µm.

In order to evaluate the presence of 5-HT, TPH and MAO A in the different stages in the bat mammary gland we realized immunofluorescence staining. Figure 3A shows a photomicrograph of bat brain tissue used as a positive control for this technique in which Raphe nuclei neurons containing 5-HT vesicles distributed in the cytoplasm are visible. The inset of Figure 3A shows a negative control of mammary gland tissue in which the slides were incubated with pre-immune serum. Figures 3B (lactation), 3C (involution) and 3D (resting phase) show MAO A immunostaining in representative mammary gland longitudinal sections, in which a positive signal was found in the myoepithelial cells (arrows), the epithelial secretory cells surrounding the alveoli (luminal), and the secretion products. Interestingly, we found differences in the quantity of the positive fluorescence signal of this enzyme in the three different stages: they were abundantly expressed during lactation; a reduction of immunostaining was apparent during involution; and scarce fluorescence appeared during the resting phase (arrow). The same pattern was observed for 5-HT (data not shown), while for TPH immunostaining we found no differences among the three physiological conditions of the mammary gland (data not shown).

### Co-localization of Serotonin/TPH and MAO A/TPH

**Figure 4:**
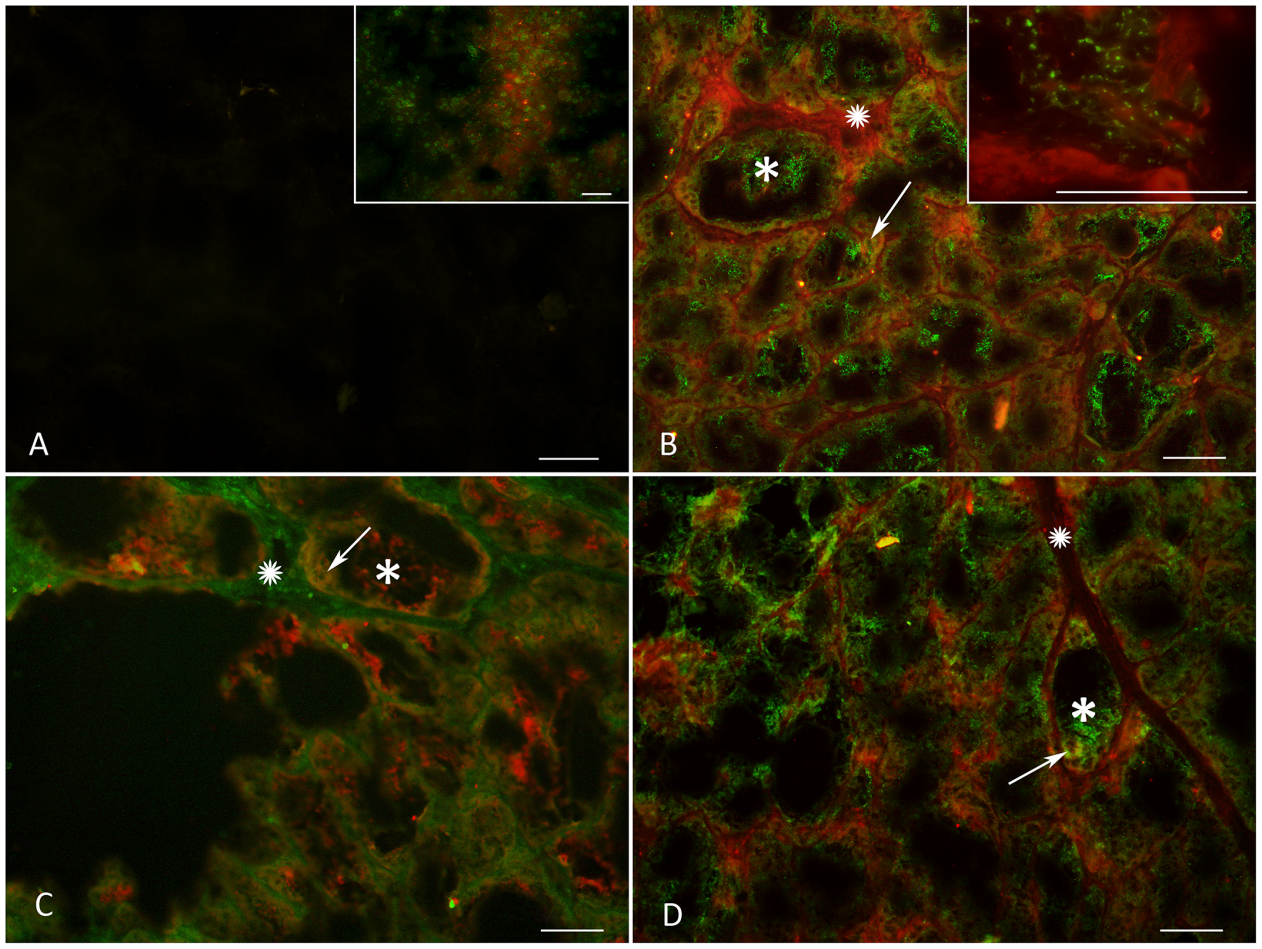
Localization of MAO A, TPH and 5-HT. Photomicrographs showing the co-localization of MAO A (green) and 5-HT (red) during lactation (4B and inset) and involution (4D). TPH (green) and MAO A (red) are shown in 4C. Figure 4A shows the negative control in which the primary antibody was omitted. The inset in Figure 4A shows a slice of the brain stem of *M. velifer* as a positive control. Transverse sections. Scale: 100 µm.

**Figure 5:**
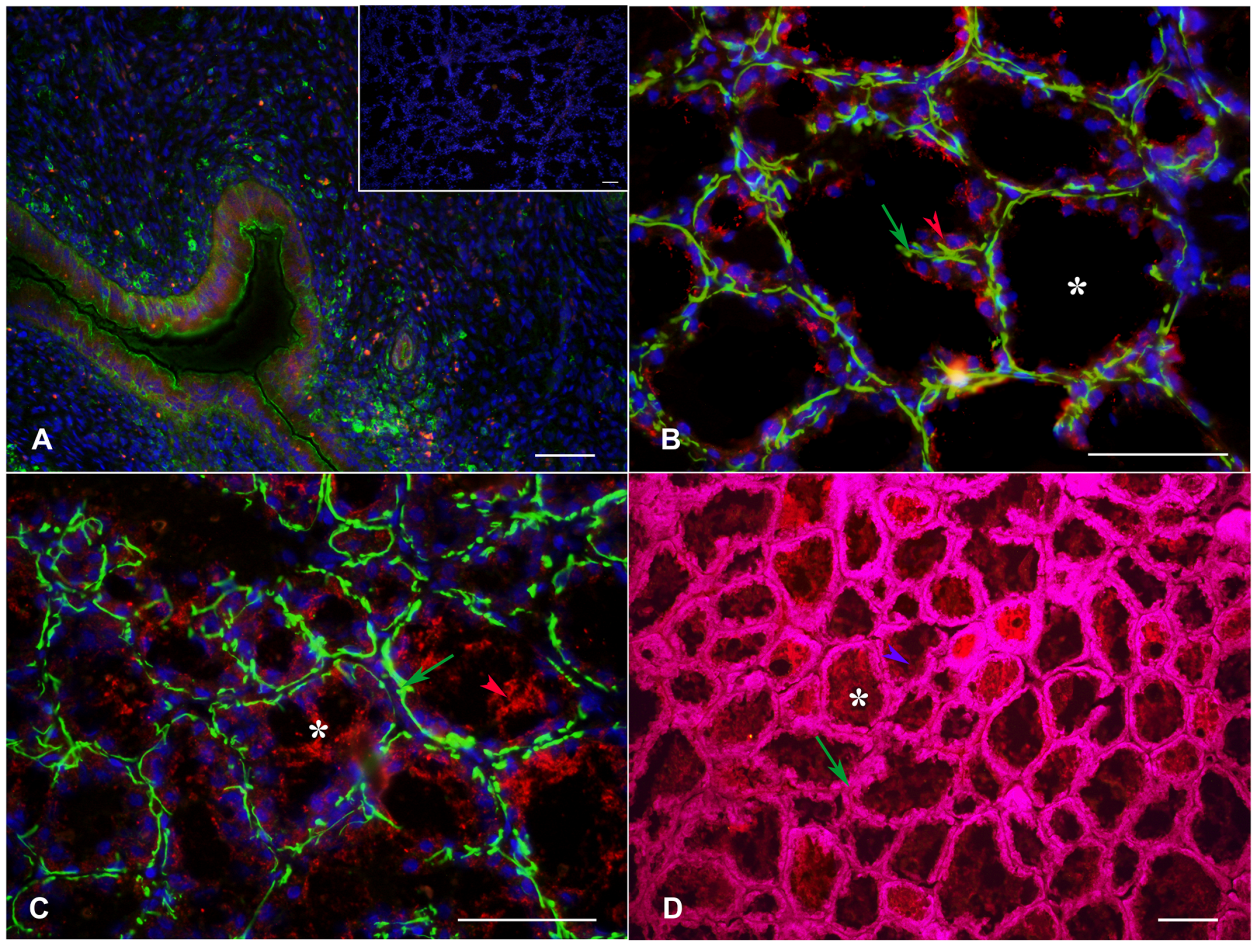
Localization of MAO A and TPH enzymes in myoepithelial and luminal epithelial cells. Photomicrographs showing the localization of TPH and MAO A in luminal epithelial and myoepithelial cells of the mammary glands from lactating females. Figure 5A shows a section of rat uterus in estrus as a positive control and the negative control of mammary gland tissue in which incubation with primary antibody was omitted (inset). Figure 5B shows the co-localization of TPH (red, red arrow) in myoepithelial cells in yellow (green arrow), while Figure 5C shows the localization of MAO A (red arrow) and myoepithelial cells in green (green arrow). Figure 5D shows the co-localization of MAO A (purple arrow) in luminal epithelial cells in magenta (green arrow). Transversal sections. Scale: 100 µm.

Once it was determined that 5-HT, TPH and MAO A were present in the mammary gland tissue, double immunofluorescence experiments were performed to further evaluate the distribution of these serotonergic markers. Positive fluorescence for 5-HT and TPH was found in the stroma (both vascular and non-vascular elements) and the epithelial cells surrounding the alveoli, while the expression of MAO A was found to be consistent in the epithelial cells and secretion products (Figure 4). Figure 4A shows a negative control in which the primary antibody was omitted. The inset in Figure 4A shows a positive control of bat brain used for this technique. Images 4B and 4D show the co-localization of 5-HT (red) and MAO A (green) in the lactation and involution phases, respectively. We found positive fluorescence for both 5-HT and MAO A in the myoepithelial and epithelial cells surrounding the alveoli (arrows) and stroma (stars), but 5-HT was absent from the secretion products in the alveoli (asterisks). Upon evaluating the interaction between these two serotonergic enzymes, TPH was found in the myoepithelial and epithelial cells (Figure 5B) of the mammary glands from lactating females, while MAO A was located in the luminal epithelial cells and secretion products (Figure 5D), but was absent in the myoepithelial cells (Figure 5C). Rat uterus tissue at estrus was used as a positive control (Figure 5A).

### Densitometry Using Immunotransference by Western Blot

Western blot analysis confirmed the presence of both enzymes TPH and MAO A in mammary gland homogenates, and showed variations of expression in the different physiological conditions of the mammary gland.

**Figure 6:**
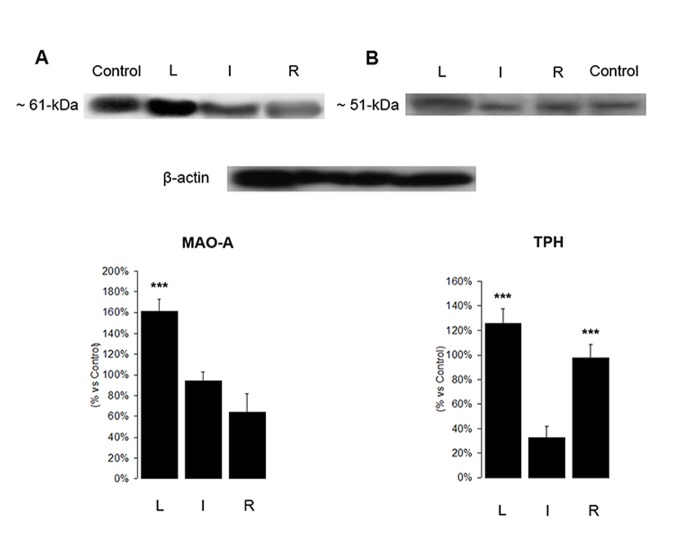
Expression of MAO A and TPH by Western blot. This image shows representative Western blots of MAO A (A), TPH (B) and β-actin as a control. Densitometry analysis for MAO A and TPH are shown; one-way ANOVA followed by a Tukey post hoc test; p<0.0001 for MAO A and p = 0.0005 for TPH. Asterisks denote statistical significance between groups.

Adult brain tissue from *M. velifer* was used as a positive control, and MAO A was identified as a band with a molecular weight of 61 kDa (Figure 6A), while TPH was identified predominantly as a single band with a molecular weight of approximately ∼51 kDa (Figure 6B). β-actin was used as a protein-loading control.

Densitometry analysis revealed differences in the intensity of the immune-stained bands in the different physiological conditions of the mammary gland. The expression of MAO A was higher during lactation and decreased in involution, while the resting phase showed the lowest expression. In addition, TPH had a significantly lower expression during involution, compared to the lactation and resting phase conditions (one-way ANOVA: p<0.0001 for MAO A and p = 0.0005 for TPH followed by a Tukey test).

### Variations in the Concentration of L-Tryptophan and 5-HT by HPLC

**Figure 7:**
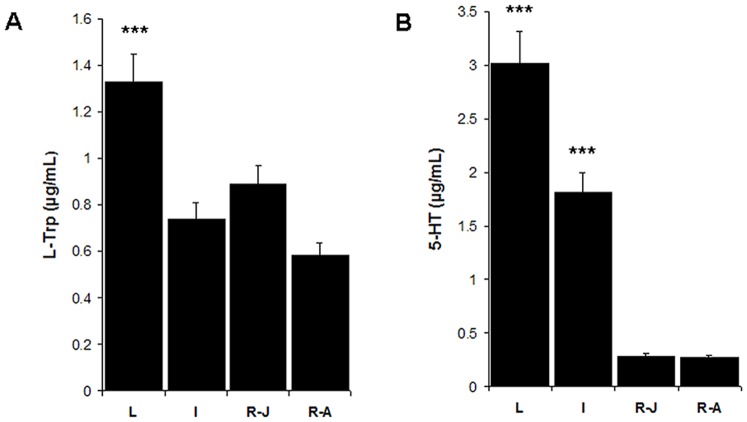
Quantitative analysis of 5-HT and L-Trp by HPLC. This image shows variations in the concentration of L-Trp and 5-HT during the different lactation stages: lactation (L), involution (I), ending of involution-beginning of the resting phase in late July (R-J), and the resting phase in August (R-A). The highest concentrations of both L-Trp and 5-HT were detected in the lactation phase; one way ANOVA followed by a Tukey post hoc test; p<0.0001. Asterisks denote statistical significance between groups.

The HPLC method was used to quantify the concentrations of L-Tryptophan (L-Trp) and 5-HT in order to evaluate the levels of both molecules in the mammary gland in the different periods during lactation. Figure 7 shows variations in the concentrations of L-Trp and 5-HT during lactation (L), post-lactation (P-L), onset of the resting phase in late July (R-J) and the resting phase in August (R-A). Both L-Trp and 5-HT were found to be abundant during lactation; however, no significant differences were found in 5-HT levels between P-L and the resting phase. The lowest levels were found in the resting phase. Significant differences were found in L-Trp, but not 5-HT, concentrations upon comparing the R-J and R-A stages (one-way ANOVA: p<0.0001 for L-Trp and 5-HT, followed by a Tukey test).

## Discussion

As it is the only organ that undergoes most of its development postnatally, the mammary gland is a highly-specialized skin gland that provides the sole food source for newborns [6]. Lactation is a complex physiological condition that induces important changes in the mammary gland; changes that are quite dramatic and can be perceived by changes in nipple size, the degree of cornification and external appearance.

Krutzsch [17] reports that the cyclical reproductive pattern of *M. velifer* is characterized by fall proestrous and mating, followed by winter hibernation with continued estrous, spring ovulation and fertilization. According to this cycle, mammary gland development begins in early spring with implantation and reaches full development in May, when parturition takes place. Krutzsch [17] found lactating females from May to early July for the populations of *M. velifer* in the southern United States; however, the physiological condition of the mammary gland was not confirmed by histology. For the populations of *M. velifer* in central Mexico, we found lactating females as early as the first week of May, and as late as the last week of July; a finding that suggests a significant variation in the breastfeeding period among individuals in these populations and, in all probability, a small variation in the lactation period between the populations of *M. velifer* in the southern U.S. and central Mexico due to geographical conditions [36]. Thus, we confirmed that lactation takes place from May to July, while the process of involution of the gland begins in early August.

Few studies of the bat mammary glands have been conducted, as most research has focused on analyzing milk composition and nursing behavior [37], [38]. One of the few histological studies was done by [38], in which the mammary gland structure of the phyllostomid bat *Carollia perspicillata* was compared to that of the mouse *Mus musculus*. Those authors report that the active mammary gland of the mouse contained a larger amount of white adipose tissue and little fibrous connective tissue in the fat pad, compared to the mammary gland of the bat. We observed a similar morphology in the mammary gland of *M. velifer* compared to that of the *C. perspicillata* bat, though they belong to different families. The facts that *M. velifer* females possess only one pair of breasts, since they are monoestrous, have a relatively long gestation period, and experience a marked development of the mammary gland during lactation, make this species a useful model for the study of the control of mammary gland development, especially with respect to control of the lactation function. Thus, research directed towards gaining a better understanding of the physiological regulation of the bat’s mammary gland, which features a recrudescence-retrogression cycle, could provide relevant information concerning the health of human subjects in relation, for example, to mammary cancer.

Recent studies show that 5-HT, along with the receptor 5-HT_7_, plays a major role in the intraluminal volume homeostasis of several organs, including the mammary gland, by functionally inhibiting milk protein synthesis and initiating the involution process [12]. During involution, 5-HT acts inside the mammary gland by indirectly promoting the opening of the tight junctions of the epithelial cells. At lower concentrations and earlier time points, 5-HT potentiates epithelial transmembrane resistance, whereas at higher concentrations and later time points, it decreases trans-epithelial electrical resistance and disrupts the tight junctions [39].

This study describes, for the first time, the expression, localization and concentration of 5-HT in the mammary gland of a monoestrous wild species. Concentrations and activity of 5-HT and the enzymes TPH and MAO A in the mammary glands of *M. velifer* were higher during lactation. Levels of TPH were lower during involution compared to the resting phase. In contrast, MAO A and 5-HT levels decreased during involution, reaching their lowest expression in the resting phase. This suggests that high 5-HT levels are needed during lactation to strengthen the tight junctions and then cause their disruption at the end of lactation, in accordance with [38]. However, when the involution process begins, 5-HT values decrease until they reach a basal level that is maintained throughout the resting phase.

5-HT action is modulated by the balance between synthesis and degradation [40] propitiated by the TPH and MAO A enzymes. The TPH is the rate-limiting enzyme in the biosynthesis of 5-HT, while MAO A enzyme oxidatively deaminates and inactivates the excess of 5-HT. Our results suggest that in the early stages of lactation of *M. velifer* both processes, the degradation and synthesis of 5-HT, occur. During involution, a high degradation of 5-HT continues, but the lower levels of TPH suggest that synthesis decreases during this phase. Finally, the lower levels of 5-HT in the resting phase may be correlated with a decreased expression of MAO A.

5-HT and TPH were found to be stored in the luminal epithelial cells that surround the alveoli and myoepithelial cells, and in stromal tissue. During pregnancy, small structures in the ductal compartment agglomerate to form an expanded lobulo-alveolar compartment composed of the basal myoepithelium and the secretory luminal epithelium [41]. While the epithelial cells are capable of synthesizing and secreting milk, the myoepithelial cells contract in response to oxytocin to generate the contractile force required for milk ejection during lactation [42]. The specific function and localization of serotonin in myoepithelial cells has not yet been described; however, we suggest that this indoleamine could contribute to myoepithelial contractile force generation during lactation. Many functions of 5-HT are still unknown, as recent studies suggest that it participates not only in mammary gland homeostasis, but might also play an important role in glucose metabolism during the transition from pregnancy to lactation [43].

MAO A was detected in luminal epithelial cells and secretion products; an unexpected finding which suggests that as a component of milk it might be involved in nourishing the offspring. This is interesting because MAO A has been linked to impulsive and aggressive behavior, suggesting that inhibition of MAO A during lactation might produce a permissive increase in aggressive behavior [44], but this requires future studies on the behavior of the offspring of bat species that form maternity colonies in which pups are grouped in nurseries and compete for adult females to feed them. Thus, additional studies are needed to confirm these ideas.

In conclusion, the results of this study strongly suggest that 5-HT plays a similar role in the bat mammary gland to that reported for other species of mammals. The content of 5-HT, MAO A and TPH varies through the different stages of mammary gland development of the adult *M. velifer* bat.
